# The roles of iPLA2, TRPM8 and TRPA1 in chemically induced cold hypersensitivity

**DOI:** 10.1186/1744-8069-6-4

**Published:** 2010-01-21

**Authors:** Clive Gentry, Natalie Stoakley, David A Andersson, Stuart Bevan

**Affiliations:** 1 Wolfson Centre for Age Related Diseases, King's College London, London SE1 1UL, UK; 2 Novartis Institutes for Biomedical Research, 5 Gower Place, London WC1 6BN, UK

## Abstract

**Background:**

The cooling agents menthol and icilin act as agonists at TRPM8 and TRPA1. *In vitro*, activation of TRPM8 by icilin and cold, but not menthol, is dependent on the activity of a sub-type of phospholipase A2, iPLA2. Lysophospholipids (e.g. LPC) produced by PLA2 activity can also activate TRPM8. The role of TRPA1 as a primary cold sensor *in vitro* is controversial, although there is evidence that TRPA1 plays a role in behavioural responses to noxious cold stimuli. In this study, we have investigated the roles of TRPM8 and TRPA1 and the influence of iPLA2 on noxious cold sensitivities in naïve animals and after local administration of menthol, icilin and LPC. The roles of the channels in cold sensitivity were investigated in mice lacking either TRPM8 (*Trpm8*^-/-^) or TRPA1 (*Trpa1*^-/-^).

**Results:**

Intraplantar administration of icilin evoked a dose-dependent increase in sensitivity to a 10°C stimulus that was inhibited by iPLA2 inhibition with BEL. In contrast the cold hypersensitivities elicited by intraplantar menthol and LPC were not inhibited by BEL treatment. BEL had no effect on basal cold sensitivity and mechanical hypersensitivities induced by the TRPV1 agonist, capsaicin, and the P2X3 agonist α,β-methylene ATP. Both *Trpm*8^-/- ^and *Trpa1*^-/- ^mice showed longer latencies for paw withdrawal from a 10°C stimulus than wild-type littermates. Cold hypersensitivities induced by either icilin or LPC were absent in *Trpm8*^-/- ^mice but were retained in *Trpa1*^-/- ^mice. In contrast, cold hypersensitivity evoked by menthol was present in *Trpm8*^-/- ^mice but was lost in *Trpa1*^-/- ^mice.

**Conclusions:**

The findings that iPLA2 inhibition blocked the development of cold hypersensitivity after administration of icilin but failed to affect menthol-induced hypersensitivity agree well with our earlier *in vitro *data showing a differential effect of iPLA2 inhibition on the agonist activities of these agents. The ability of LPC to induce cold hypersensitivity supports a role for iPLA2 in modulating TRPM8 activity *in vivo*. Studies on genetically modified mice demonstrated that the effects of icilin and LPC were mediated by TRPM8 and not TRPA1. In contrast, menthol-induced cold hypersensitivity was dependent on expression of TRPA1 and not TRPM8.

## Background

TRPM8 expressed by a sub-population (~10%) of primary afferent sensory neurons has a role in the detection and transmission of cold stimuli. This channel is activated by cool temperatures with a threshold for activation in the range 20-30°C. In addition, TRPM8 is activated by the cooling compounds icilin and menthol, which shift the threshold for thermal activation to higher temperatures [1-3].

The activity of TRPM8 can also be modulated by other factors, such as the binding of phosphatidylinositol 4,5-bisphosphate (PIP2) and membrane depolarization [1-5]. Our earlier studies also demonstrated that endogenous lysophospholipids (LPLs) generated by the calcium-independent form of the enzyme phospholipase A2 (iPLA2) regulated TRPM8 activity [6].

Another TRP channel, TRPA1, is expressed in about half of the sensory neurons that express TRPV1 and therefore is associated with nociceptive responses. Some publications have linked TRPA1 expression with the ability to sense cold pain, although the ability of TRPA1 to respond directly to cold temperatures is controversial [7-9]. Not all studies found that TRPA1 could be activated by cold [10-13], although some recent publications have provided support for a role in cold transduction *in vitro *[14-17] and *in vivo *[15,18].

The cooling agent, icilin, activates both TRPM8 [2,19,20] and TRPA1 [21]. Systemic administration of icilin produces behaviours such as wet dog shakes and jumping in rodents that are absent in *Trpm8*^-/- ^mice [22,23]. Menthol also activates TRPM8 and TRPA1 at similar concentrations, although at higher concentrations it blocks the activity of rodent TRPA1 channels [24-26]. Topical application of menthol to healthy human volunteers sensitizes the oral responses to innocuous cold temperatures and skin responses to noxious cold stimuli [27-31].

There are conflicting data about the contribution of TRPM8 to cold withdrawal responses, with reports that *Trpm8*^-/- ^mice either have the same withdrawal latencies as wild type mice from a -1°C to 0°C cold plate [32,33] or show a reduced cold sensitivity [23]. Injection of icilin into the paw reduces cold plate paw withdrawal latency in wild type mice but not in *Trpm8*^-/- ^mice [32] consistent with a major role of TRPM8 in icilin-induced cold hypersensitivity. Whether icilin activation of TRPA1 can also induce cold hypersensitivity is unknown. The contributions of TRPM8 and TRPA1 to menthol-induced cold hypersensitivity are also unknown.

Earlier studies showed that the activity of TRPM8 was modulated by the activity of a sub-type of phospholipase A2, namely iPLA2 [6,34]. An iPLA2 inhibitor (bromenol lactone, BEL), abolished the response of TRPM8 to icilin, reduced the cold sensitivity of the channel and abolished the responses to cold stimuli in the majority of cold-sensitive dorsal root ganglion neurons. A reduction of iPLA2 expression with antisense oligonucleotides has also been shown to inhibit TRPM8 activity [34]. Furthermore lysophospholipids (e.g. LPC), which are the products of PLA2 activity, raised the temperature threshold for TRPM8 activation towards normal body temperature and so stimulated channel activity at experimental temperatures above 30°C [6].

In the current series of *in vivo *experiments in rats we have examined the effects of inhibiting iPLA2 on noxious cold responses in naïve animals and after intraplantar administration of icilin and menthol, and investigated the effects of local administration of LPC on noxious cold responses. We have also used genetically modified mice lacking either TRPM8 or TRPA1 to probe the contribution of these channels to the development of cold hypersensitivities evoked by these agents. Our data demonstrate that iPLA2 activity is required for icilin-induced cold hypersensitivity, which is mediated exclusively by TRPM8, and that LPC-induced increases in cold sensitivity depend on the presence of TRPM8. Menthol-induced cold sensitivity was not affected by iPLA2 inhibition and was still evident in TRPM8-deficient mice. The effect of menthol on cold-hypersensitivity was, however, absent in mice lacking TRPA1, indicating that activation of TRPA1 and not TRPM8 is responsible for the observed behavioural responses to noxious cold after administration of menthol.

## Results

### Measurement of cold sensitivity

Two methods were initially used to assess cold sensitivity using a temperature controlled metal plate. First, the time to the first behavioural response (paw lick/lift) in freely moving rats. Second, the time taken for lightly restrained rats to withdraw their paw from the cold plate.

**Figure 1:**
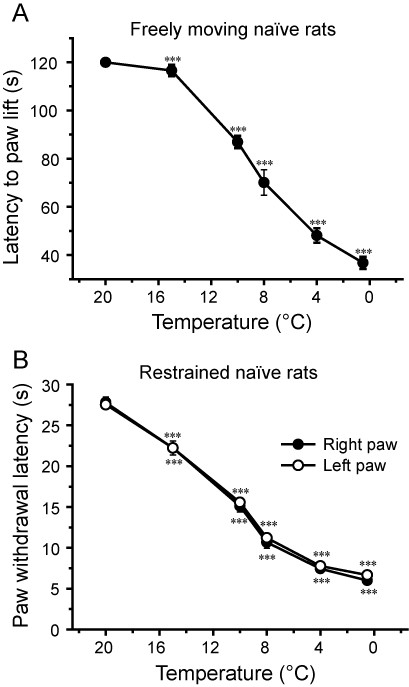
**Temperature dependence of withdrawal latencies**. **A**. Response time for rat hind-limb paw withdrawal (lick or lift) from a cold plate set at various temperatures for unrestrained, freely moving rats. **B**. Paw withdrawal latencies for left and right hind-limbs in lightly restrained rats. Note the similar results obtained with these two methods and the close correspondence between the values for left and right limbs in restrained animals. Data shows mean ± SEM for 9 rats.*** p < 0.001 vs. 20°C readings.

The responses to varying cold stimuli were first investigated in untreated rats using a range of cold-plate temperatures from 0.5°C to 20°C (Figure 1A). The paw withdrawal latencies decreased with reduced cold-plate temperatures with a mid point at about 10°C. This temperature was used in subsequent experiments as it was suitable to show either an increase or a decrease in cold sensitivity. Similar results were obtained with freely moving and restrained animals, but one major advantage of using the held animals is that readings for both the left and right hind paws can be obtained; this is not easily achieved using freely moving animals. As shown in Figure 1B almost identical paw withdrawal latencies were obtained for the left and right paws. Because of this added advantage and the consistency of the data obtained, the restrained method was used in most of the studies.

### Effects of iPLA2 inhibition on cold sensitivity

**Figure 2:**
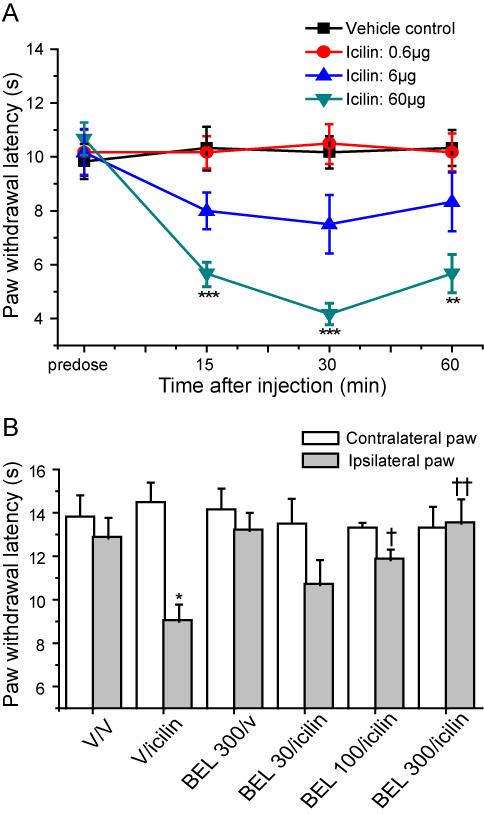
**Icilin-induced cold hypersensitivity is inhibited by the iPLA2 inhibitor, BEL**. **A**. Ipsilateral paw withdrawal latencies in lightly restrained rats at various times after intra-plantar administration of 0.6, 6 or 60 μg icilin or vehicle alone. **B**. Prior administration of 30-300 μg BEL dose dependently inhibited the cold hypersensitivity induced by 60 μg icilin. Data show mean ± SEM for 6 rats/group. * p < 0.05, ** p < 0.01, *** p < 0.001 vs vehicle. In B, † p < 0.05, †† p < 0.01 when compared with vehicle/icilin treated group.

Earlier *in vitro *experiments showed that iPLA2 inhibition abolished the responses of TRPM8 to icilin but not to menthol [6]. The effects of iPLA2 inhibition on icilin-induced cold hypersensitivity were therefore investigated to determine if this dependence on iPLA2 was present *in vivo*. Intra-plantar injection of icilin elicited a dose-related increase in cold sensitivity that was stable from 15 to 60 minutes following administration. At 6 μg and 60 μg there was a marked reduction in the paw withdrawal latency (Figure 2A). Doses above 60 μg were not used as they induced side effects, notably characteristic 'wet dog shakes' [35,36]. Prior administration of the selective iPLA2 inhibitor BEL (30-300 μg) had no effect on baseline cold sensitivity (data not shown) but inhibited the icilin-induced cold sensitivity significantly at 100 and 300 μg in a dose-dependent manner (Figure 2B).

**Figure 3:**
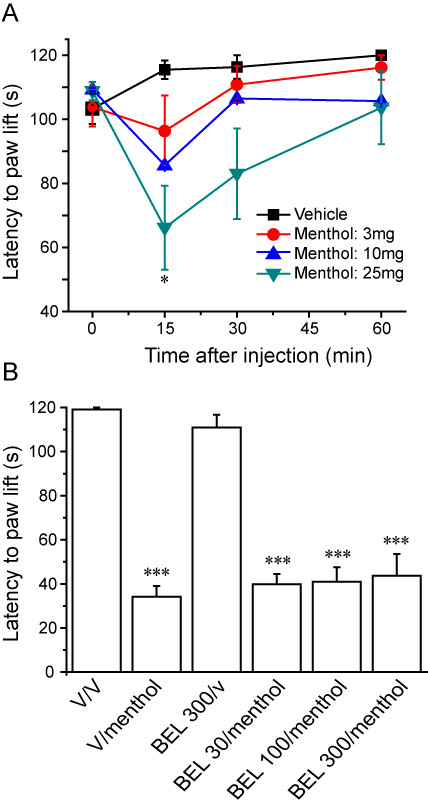
**Menthol-induced cold hypersensitivity is not inhibited by BEL**. **A**. Intraplantar administration of menthol (3-25 mg) induced a time- and dose-dependent reduction in ipsilateral paw withdrawal latency in freely-moving rats. **B**. Prior administration of BEL (30-300 μg) had no effect on the cold hypersensitivity evoked by 25 mg menthol. Data show mean ± SEM for 6 rats/group. * p < 0.05, *** p < 0.001 vs vehicle.

A second set of experiments was performed in rats to investigate the effects of iPLA2 inhibition on menthol-induced cold hypersensitivity. Intra-plantar injection of menthol led to a dose-dependent cold hypersensitivity. Figure 3A illustrates the results of an experiment in freely moving rats where 25 mg menthol, which was the highest concentration that we could use, gave the maximal observed effect. Lower doses of menthol (10 mg and below) produced less consistent results. Menthol (2.5-25 mg) also evoked cold hypersensitivity in restrained animals although with this method a similar degree of hypersensitivity was noted for the 8.3 mg and 25 mg doses (Additional file 1). No wet dog shakes were evoked by menthol. In contrast to the effects on icilin-induced cold hypersensitivity, prior administration of BEL (30-300 μg) had no effect on the cold sensitivity induced by menthol (Figure 3B).

### Effects of LPC on cold sensitivity

**Figure 4:**
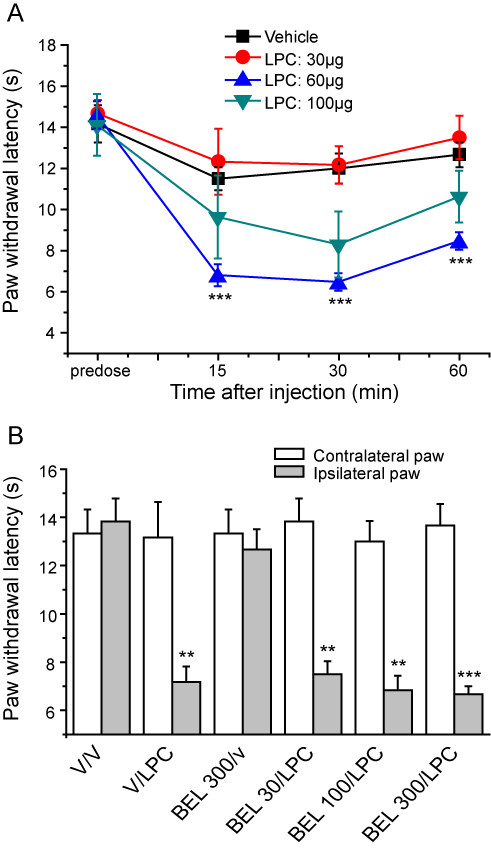
**LPC-induced cold hypersensitivity is not inhibited by BEL**. **A**. Intraplantar administration of LPC evoked a dose-dependent cold hypersensitivity in lightly restrained rats. **B**. Prior administration of BEL (30-300 μg) did not inhibit the cold hypersensitivity evoked by 60 μg LPC. Data show mean ± SEM for 6 rats/group. ** p < 0.01, *** p < 0.001 vs vehicle.

Lysophospholipids, such as LPC, sensitize TRPM8 *in vitro *and raise the thermal threshold towards body temperature [6]. We therefore investigated whether or not local administration of LPC would increase cold sensitivity *in vivo*. Intra-plantar injection of LPC (30-100 μg) resulted in an increased cold sensitivity as shown by a reduction in paw withdrawal latency (Figure 4A). A marked cold hypersensitivity was evident with 60 μg LPC but not with the 30 μg dose. The hypersensitivity produced by 100 μg LPC was less than at 60 μg. One possible explanation for this finding is that LPC exhibits a bell-shaped dose-response, but it is equally likely that poor solubility at higher concentrations is an issue. When tested in a hot-plate latency test using a 50°C stimuli no significant increase in heat sensitivity was seen following 60 μg LPC injection (Additional file 2).

Lysophospholipids are produced by the activity of PLA2 and so PLA2 inhibition would not be expected to affect any TRPM8-mediated behavioural effects of LPC. This was examined by pre-treating rats with an intraplantar injection of BEL (30-100 μg) to inhibit iPLA2 prior to local administration of 60 μg LPC. BEL had no effect on the development of LPC-induced cold hypersensitivity as the paw withdrawal latencies were very similar in vehicle and BEL pre-treated groups (Figure 4B). No significant changes in the contralateral paw withdrawal latencies were noted in these experiments (data not shown).

### Selectivity of the iPLA2 pathway

In order to gain information about the selectivity of the iPLA2 pathway for TRPM8 mediated events, we also examined the effects of BEL on hypersensitivities induced by a TRPV1 agonist, capsaicin, and a P2X3 agonist α,β-methylene ATP (α,βMeATP), in rats. Intraplantar injection of both agents induced a mechanical hypersensitivity measured by reduced paw withdrawal thresholds to mechanical pressure. Pre-treatment with intraplantar BEL (100 μg) had no effect on the development of mechanical hypersensitivities to either capsaicin or α,βMeATP (Additional file 3).

### The effects of LPC and icilin on cold sensitivity in mice lacking TRPM8 or TRPA1

**Figure 5:**
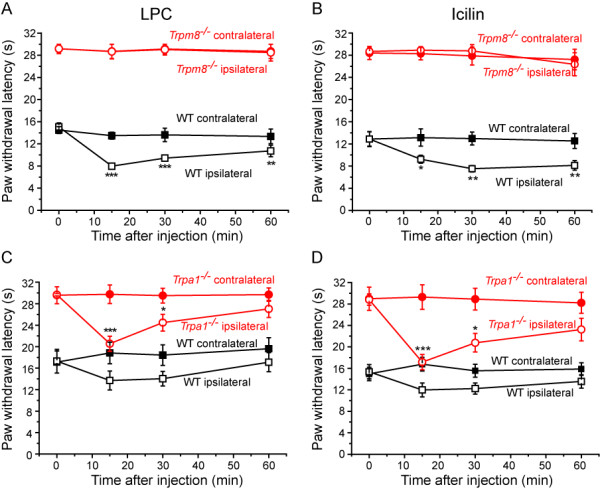
**Cold hypersensitivities evoked by icilin and LPC are absent in *Trpm8*^-/- ^mice but retained in *Trpa1*^-/- ^mice**. Effects of intraplantar administration of either 60 μg LPC (**A**) or 100 μg icilin (**B**) on cold withdrawal latencies in lightly restrained *Trpm8*^-/- ^and wild-type littermate mice. Neither compound had an effect on withdrawal latency for the injected (ipsilateral) paw in *Trpm*8^-/- ^mice. Both compounds reduced the ipsilateral paw withdrawal latencies in wild-type littermates, but had no effect on withdrawal latencies for the uninjected (contralateral) paw. Intraplantar LPC (**C**) or icilin (**D**) reduced paw withdrawal thresholds for ipsilateral paws in both *Trpa1*^-/- ^and wild-type littermate mice. Note that a greater cold hypersensitivity was seen in *Trpa1*^-/- ^than in wild-type mice. Data show mean ± SEM for 6 mice/group. * p < 0.05, ** p < 0.01, *** p < 0.001 vs relevant predose values. † p < 0.05, †† p < 0.01 for contralateral-ipsilateral difference in wild-type mice.

The role of TRPM8 in mediating LPC evoked cold hypersensitivity was investigated in mice, taking advantage of the availability of genetically modified animals lacking TRPM8. The paw withdrawal latency of *Trpm8*^-/- ^mice to a cold stimulus was longer than that in wild-type littermates (Figure 5A, B). Intraplantar injection of LPC in wild-type mice evoked a cold hypersensitivity as seen previously in rats. In contrast, LPC failed to induce cold hypersensitivity in *Trpm8*^-/- ^mice (Figure 5A). The effects of intraplantar injections of icilin were also evaluated in *Trpm8*^-/- ^mice and wild-type littermates. Icilin induced a cold hypersensitivity in wild-type mice but not in *Trpm8*^-/- ^mice (Figure 5B).

As cold sensitivity *in vivo *is also influenced by another sensory neuron TRP channel, TRPA1, we also investigated the effects of LPC in mice lacking TRPA1. The basal cold threshold was higher in *Trpa1*^-/- ^mice than in wild-type littermates (Figure 5C, D) as has been shown previously for this line of genetically modified mice [15,18,37]. Nevertheless, intraplantar injection of LPC was able to induce cold hypersensitivity in both groups of mice illustrating that the effects of LPC are independent of TRPA1 (Figure 5C). Similarly icilin-induced cold hypersensitivity was retained in *Trpa1*^-/- ^mice. Icilin induced a modest cold hypersensitivity in wild-type mice but a marked reduction in withdrawal latency was noted in *Trpa1*^-/- ^mice indicating no loss of induced cold hypersensitivity (Figure 5D).

### Menthol-induced cold hypersensitivity is dependent on TRPA1 but not TRPM8

**Figure 6:**
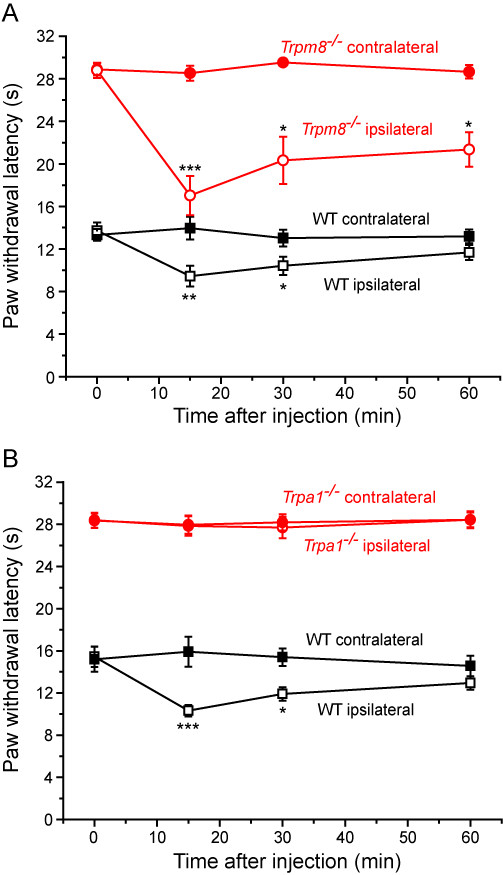
**Menthol-evoked cold hypersensitivity is absent in *Trpa1*^-/- ^mice but present in *Trpm8*^-/- ^mice**. **A**. Intraplantar administration of 25 mg menthol evoked marked cold hypersensitivity in the ipsilateral paw of lightly restrained *Trpm8*^-/- ^mice. Note that a greater cold hypersensitivity was seen in *Trpm8*^-/- ^than in wild-type mice. **B**. Menthol evoked cold hypersensitivity in wild-type but not *Trpa1*^-/- ^mice. Data show mean ± SEM for 6 mice/group. * p < 0.05, ** p < 0.01, *** p < 0.001 vs relevant predose values. † p < 0.05, †† p < 0.01, ††† p < 0.001 for contralateral-ipsilateral difference in wild-type mice.

The failure of BEL to inhibit menthol induced cold hypersensitivity was consistent with our *in vitro *data showing that BEL did not inhibit the agonist effects of menthol on TRPM8 [6]. As menthol can also activate TRPA1 [24-26], we also examined if the observed behavioural effect of menthol could be mediated by TRPA1 and not by TRPM8. Menthol was administered by intraplantar injection in *Trpm8*^-/- ^and *Trpa1*^-/- ^mice and their respective wild-type littermates. Menthol evoked cold hypersensitivity in both wild-type and *Trpm8*^-/- ^mice (Figure 6A). In contrast, menthol induced cold hypersensitivity in wild-type (*Trpa1*^+/+^) mice, but had no significant effects on cold thresholds in *Trpa1*^-/- ^mice (Figure 6B). The vehicles used in these experiments had no effect on the behavioural responses at any temperature. Both icilin and menthol do induce initial pain responses at room temperature (paw licking, biting and shaking) but these effects were short-lived (less than 10 minutes) and were not evident at the times used to assess the evoked responses to thermal stimuli.

Given the failure of menthol to elicit cold hypersensitivity in *Trpa1*^-/- ^in the cold plate assay, we also assessed the role of TRPA1 using an acetone evaporation method. Acetone administration evoked a significant behavioural response in *Trpa1*^+/+^mice which was greatly diminished in *Trpa1*^-/- ^mice. Following intraplantar administration of menthol the acetone evoked paw responses were increased in wild-type *Trpa1*^+/+^mice but not in *Trpa1*^-/- ^mice (Additional file 4).

**Figure 7:**
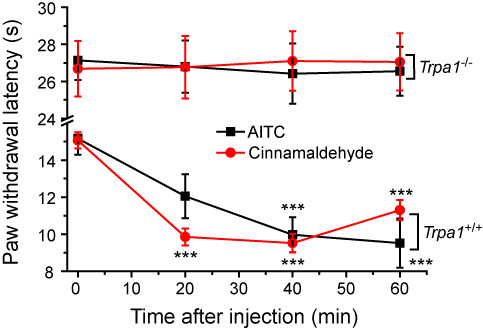
**TRPA1 agonists AITC and cinnamaldehyde evoke cold hypersensitivity**. Intraplantar injection of either AITC (100 μg) or cinnamaldehyde (50 μg) evoked marked cold hypersensitivity in the ipsilateral paw of lightly restrained wild-type mice but had no effect when administered to *Trpa1*^-/- ^mice. Data show mean ± SEM for 6 mice/group. *** p < 0.001 vs pre-dose latencies.

Other TRPA1 agonists were also tested in the cold-plate assay to determine if cold hypersensitivity was a general feature of TRPA1 agonism. Both allyl isothiocyanate (AITC, 100 μg i.pl.) and cinnamaldehyde (50 μg i.pl.) induced a significant cold hypersensitivity in the injected paw of wild-type (*Trpa1*^+/+^) mice but had no effect in *Trpa1*^-/- ^mice (Figure 7). No significant changes in cold evoked withdrawal latencies were noted in the contralateral paws (data not shown) of wild-type mice, which demonstrated that there were no systemic effects of locally administered AITC and cinnamaldehyde.

## Discussion

The methods used to assess the behavioural changes to a cold stimulus vary widely in different laboratories. Methods described in the literature include observations of freely moving animals on a cold-plate or in a water bath, the latencies for tail or paw withdrawal from cold water using restrained animals and behavioural responses induced by acetone evaporation. Furthermore, the temperatures used differ considerably and are often overtly noxious. The use of freely moving animals on a cold-plate has limitations, particularly where mice are concerned as good responses are not readily obtained [38]. In addition it is difficult to evaluate the responses of each hind paw to the stimulus in freely moving animals. This precludes ready comparison of the effects of local administration of compounds into the ipsilateral (treated) and contralateral (untreated) limbs. Also, prolonged whole body exposure to cold in animals moving around on a cold plate may influence the behavioural response by stimulating higher brain centres rather than reporting peripheral transduction mechanisms. Such whole body exposure to cold can also cause the animals to become less mobile rather than evoking a measurable nociceptive response. In contrast, measurements of responses in lightly restrained animals allowed cold sensitivity to be evaluated in both paws in individual animals using a local cold stimulus. With the plate set at ambient temperatures (25-37°C), no paw withdrawals were observed with the 30 second cut-off used in these studies. In other experiments we noted that most animals did not withdraw their paws at ambient temperature during a 60 second observation period. It is therefore unlikely that simple mechanical sensitivity accounts for the paw withdrawal. In the traditional cold-plate assay, freely roaming rodents walk on the plate and do not usually have a single paw in constant contact with the cold-plate. Our method using lightly restrained animals provides continuous contact between the paw and the cold-plate, which results in shorter withdrawal threshold latencies and less variable responses.

Our finding of an increased latency for paw withdrawal from a 10°C cold plate in *Trpm8*^-/- ^mice supports a role for TRPM8 in the behavioural responses to cold. This result is consistent with the findings of one earlier study [23], but disagrees with results from two other laboratories [32,33]. One of the studies that did not show an increased latency in *Trpm8*^-/- ^mice [32] used a lower cold-plate temperature (-1°C) than in the current investigation. In the other study, Bautista et al. [33] did not observe a difference in cold-plate responses in freely moving *Trpm8*^-/- ^and wild-type mice over a range of temperatures (-10°C - 10°C) including the temperature (10°C) used in our studies. We can rule out the possibility that differences in mouse strains were responsible for the different results as the mice used in our experiments were from the same stock as those used by Bautista et al. [33]. Both published studies did, however, detect a reduced behavioural response in *Trpm8*^-/- ^mice in acetone-induced paw flinching assays consistent with a reduction in cold sensitivity [32,33]. The reason for the differences in cold-plate results between studies is unclear. It is possible that the less variable latencies that we observe with restrained mice with their paws in constant contact with the cold surface have allowed us to detect a reduced cold-sensitivity in the *Trpm8*^-/- ^mice. Such a cold-response deficit is consistent with the findings of Colburn et al [23] who showed a marked (three-fold) increase in cold-plate response latencies in *Trpm8*^-/- ^mice.

The results of the *in vivo *studies with icilin and LPC agree well with our earlier *in vitro *studies [6] demonstrating that LPLs produced by iPLA2 play an important role in regulating the cold sensitivity of TRPM8. iPLA2 inhibition with BEL reduced the cold hypersensitivity induced by icilin, consistent with our *in vitro *findings that BEL inhibited TRPM8 responses to icilin. These results suggest that icilin activation of TRPM8 requires a product of iPLA2 activity perhaps acting as a cofactor that enables activation by icilin. Icilin acts as an agonist at both TRPM8 and TRPA1 channels. Our behavioural data with *Trpm8*^-/- ^and *Trpa1*^-/- ^mice show that icilin induction of cold hypersensitivity was dependent on the presence of TRPM8, in agreement with the findings of Dhaka et al. [32], and was not influenced by the presence or absence of TRPA1.

The finding that BEL treatment did not affect the baseline responses to the cold (10°C) stimulus indicates that tonic activity of iPLA2 does not contribute to the behavioural responses to noxious cold under normal circumstances. It is possible that the level of tonic iPLA2 activity is usually low and therefore no baseline modulation of TRPM8 activity occurs when the enzyme is inhibited. Such a conclusion would suggest that icilin stimulates iPLA2 activity either directly or indirectly.

The ability of LPC to induce cold hypersensitivity *in vivo *supports a role for iPLA2 in modulating TRPM8. The absence of any effect on the sensitivity to a noxious heat stimulus indicates that this is not due to general neuronal sensitization. The experiments with *Trpm8*^-/- ^mice showed that LPC-induced cold hypersensitivity was dependent on the presence of TRPM8 and was not due to an action of LPC on other targets. The lack of effect of iPLA2 inhibition by BEL on cold hypersensitivity induced by LPC was expected as LPC is the product of this enzyme. This result, together with the findings that BEL did not influence the mechanical hypersensitivities induced by intraplantar injection of capsaicin or the P2X3 agonist, α,βMeATP, show that BEL does not have non-specific effects on nociception in primary afferent neurons. The lack of effect of BEL on chemically evoked mechanical sensitivity indicates that the compound had no significant inhibitory actions on either activation of TRPV1 or P2X3 receptors by their respective agonists or the transduction and transmission of noxious mechanical stimuli.

Although there has been some debate concerning the role of TRPA1 in cold transduction [7], the behavioural data in our current study and in some other investigations on *Trpa1*^-/- ^mice [15,18,37] show that mice lacking TRPA1 have reduced behavioural responses to cold. Other investigators have failed to show reduced cold sensitivity in *Trpa1*^-/- ^mice [39]. Mechanisms, such as TRPM8 activation or cold inhibition of a potassium conductance [40,41] in sensory neurons, also contribute to cold responses. Cold sensation *in vivo *may depend on a number of mechanisms with varying contributions at different temperatures.

The finding from experiments in genetically modified mice that menthol-induced cold hypersensitivity was not obviously influenced by TRPM8 expression but was dependent on TRPA1 expression was initially surprising. The influence of TRPA1 on the development of menthol-induced cold hypersensitivity was therefore confirmed using a different method, measuring the behavioural responses to acetone evaporation. Menthol is a good TRPM8 agonist and so a TRPM8 mediated menthol-induced cold hypersensitivity would be expected in the *Trpa1*^-/- ^mice, similar to that seen for LPC. Menthol can, however, affect some other ion channels which may contribute an inhibitory component to its overall effect. For example, at higher concentrations menthol directly activates GABA_A _receptors [42] and has an inhibitory effect on T-type Ca channel activity [43]. Both these actions could inhibit sensory neuron activation and firing. In the absence of an excitatory input from TRPA1 expressing neurons, an inhibitory effect of menthol via these other mechanisms may be sufficient to block the menthol-evoked excitatory input from cold-sensitive TRPM8 neurons. The concentrations of menthol that activate TRPM8 and TRPA1 *in vitro *are very similar [24,25] and it is unlikely that menthol would selectively activate TRPA1 *in vivo*. High concentrations of menthol can inhibit TRPA1 *in vitro *[24-26]. However, the highest concentration of menthol that we could use (25 mg) evoked cold hypersensitivity in wild-type rats and mice and *Trpa*1^-/- ^mice, so it is unlikely that we achieved inhibitory concentrations of the compound *in vivo*.

LPC and icilin induced a greater cold hypersensitivity in *Trpa1*^-/- ^than in the WT mice. It is possible that the presence of TRPA1 could in some way reduce the hypersensitivity induced by these agonists, although there have been no reports of a tonic analgesic effect mediated by TRPA1. Another possibility is that the stimulation of a TRPM8-linked pathway by agonists is enhanced in the absence of TRPA1. We also found that menthol induced a greater cold hypersensitivity in *Trpm8*^-/- ^mice than in WT mice. Activation of TRPM8 has been shown to induce centrally-mediated analgesic effects [44] and it is possible that the absence of a component of TRPM8 mediated analgesia contributes to the larger induced cold hypersensitivity. However, the elevated baseline latencies seen with both *Trpm8*^-/- ^and *Trpa1*^-/-^mice may provide a common and simpler explanation as there is a greater range for reductions in paw withdrawal latencies in the gene deficient mice.

While TRPA1 may or may not act as a direct sensor of noxious cold temperature *in vitro *[8,9,15,16], the evidence from the studies reported here and elsewhere [15,45] indicates that *in vivo *TRPA1 does influence the behavioural responses to noxious cold. Our data also reinforce the need for caution in the interpretation of data obtained using menthol as an agonist *in vivo *as the effects of this agent can be due to an action on TRPA1 rather than TRPM8.

## Methods

### Animals

All animal studies were performed according to the UK Home Office Animal Procedures Act (1986) after in-house ethical review.

Adult male Wistar rats, weighing approximately 180-200 g were purchased from Harlan (Bicester, Oxon, U.K.). TRPA1-null mice and wild-type littermates were bred from heterozygotic mice kindly provided by Drs. Kelvin Kwan (Harvard Medical School, Boston, MA) and David Corey (Harvard Medical School, Boston, MA) [18]. TRPM8-null mice and wild-type littermates were bred from heterozygotic mice kindly provided by Dr. David Julius (University of California, San Francisco, California) [33].

### Drug administration

Injections (25 μl) were made subcutaneously into the plantar surface of one of the hind paws using a 50 μl luer-syringe (Hamilton, Reno, NV) fitted with a 26-gauge by 3/8 inch intradermal needle. Icilin (Biomol International) was dissolved in DMSO and diluted to 50% in saline. Menthol and 1-palmitoyl-sn-glycero-3-phosphocholine (LPC, Sigma, Poole) were dissolved in saline and bromoenol lactone (BEL, Sigma) was made up in DMSO. The vehicle for capsaicin (Sigma) was 10% DMSO in saline and α,β-methylene ATP (α,βMeATP, Sigma) was dissolved in saline. A stock solution of AITC was made up in 50%ethanol/10% Tween 80/saline which was then diluted serially 100-fold in saline to obtain the final concentration. Cinnamaldehyde was diluted in 0.5% Tween 80 in saline.

Injections of vehicle solutions had no effect at room temperature. Intraplantar injections of icilin, menthol, allyl isothiocyanate and cinnamaldehyde evoked some initial pain responses (paw licking and shaking) but these effects were short lived (less than 10 minutes) and were not apparent at times when responses to thermal or mechanical stimuli were tested. A few rats exhibited characteristic 'wet dog' shakes after administration of icilin but these animals were not used in the studies reported here.

### Behavioural tests, temperature sensitivity

Cold sensitivity was assessed with a cold-plate using two different methods. Firstly, animals were placed onto the cold-plate within a perspex enclosure and the first sign of ipsilateral paw lift or lick was recorded as the paw withdrawal latency. A maximum cut-off paw withdrawal latency of 120 seconds was used to prevent possible tissue damage and unnecessary cold-induced trauma to the animal. In the second method the animals were lightly restrained and each hind paw in turn placed onto the surface of the cold-plate. The end point was taken as the withdrawal of the paw and recorded as the withdrawal latency for the ipsilateral and the contralateral paw. A maximum cut-off of 30 seconds was used for each paw. Experiments were performed using a cold-plate equipped with a Peltier and water cooled via a flow-cooler (Techne FC-200) and circulator (Techne C-85A) (Mechanical workshop, Novartis Institutes for Biomedical Research) or latterly using a commercially available cold-plate (Ugo Basile, Milan). The cold-plates were set according to pre-determined calibration data using a surface temperature probe to correlate set temperature to actual surface temperature over a wide temperature range (-5°C to 26°C). The cold plate was allowed to stabilize for 5 minutes prior to testing at each temperature.

Using both methods the paw withdrawal latencies were determined at a range of temperatures (0.5° to 20°C). For all subsequent studies the cold-plate was set at 10°C. Dose responses to icilin, LPC and BEL were obtained and doses selected for use in future studies. In iPLA2 inhibitor studies predose readings were taken prior to drug administration. BEL was then administered 1 hour before induction of cold hypersensitivity with icilin or LPC and subsequent post-dose readings were taken after 15 minutes.

In one set of experiments we also assessed cold sensitivity using acetone evaporation based on a previously published method [46]. Animals were placed into wire grid bottom cages allowing access to the underside of their paws and habituated to this environment prior to the start of the experiment. Acetone (0.05 ml) was applied to the centre of the plantar hind-paw using a micropipette and the animal's response monitored for 40 seconds. Responses to acetone were graded using the following four point scale: 0 = no response, 1 = quick withdrawal, flick or stamp of the hind-paw, 2 = prolonged withdrawal or repeated (>2) flicking of the hind-paw, 3 = repeated flicking of the paw with licking directed at the affected limb. Acetone was applied alternately three times to each hind-paw and the responses recorded. Cumulative scores were generated for each hind-paw by adding the 3 scores for each paw, the minimum score being 0 (no response to any trial) and the maximum possible score being 9 (repeated flicking and paw licking in all trials). The effect of menthol was assessed at a single time point, 15 minutes after intraplantar injection of 25 mg menthol. This time point was chosen on the basis of the time course of cold-plate hypersensitivity seen in wild-type mice.

Heat sensitivity was assessed by measuring the time for paw withdrawal in lightly restrained rats using a calibrated hot-plate (Ugo Basile, Milan) at 50°C. A maximum, cut-off paw withdrawal latency of 15 seconds was used to prevent possible tissue damage and unnecessary trauma to the rats.

### Behavioural tests, mechanical sensitivity

Mechanical sensitivity was assessed in lightly restrained rats by measuring paw withdrawal thresholds to an increasing mechanical force applied to the dorsal surface of the rat paw using an Analgesymeter (Ugo-Basile, Milan). The analgesymeter employed a wedge shaped probe (area 1.75 mm2). Cut-off was set at 250 grams and the end point was taken as withdrawal of the hind paw. Data are expressed as withdrawal thresholds in grams. Paw withdrawal thresholds were determined in the hind paws of both ipsilateral and contralateral hind limbs.

Predose readings were taken prior to drug administration BEL was then administered 1 hour before induction of mechanical hypersensitivity by the injection of capsaicin (1 nmole in 10% DMSO in saline) or α,βMeATP (1 μmole in saline). Post-dose readings were taken after 30 minutes.

### Statistical analysis

Statistical analysis was carried out on raw data using repeated measures of ANOVA followed by post-hoc analysis using Tukey's HSD test (p < 0.05 was set as the level of statistical significance).

## Competing interests

The authors declare that they have no competing interests.

## Authors' contributions

SB conceived the study, performed the mouse genotyping and drafted the manuscript. CG and NS designed and performed the *in vivo *behavioural studies; CG also helped draft the manuscript. DA participated in the study design and helped to draft the manuscript. All authors read and approved the final manuscript.

## Supplementary Material

Additional file 1 **Intraplantar administration of menthol (2.5-25 mg) induced a time- and dose-dependent reduction in ipsilateral paw withdrawal latency in restrained rats.** Data show mean ± SEM for 6 rats/group. * p < 0.05, *** p < 0.001 vs vehicle. Click here for file

Additional file 2 **Intraplantar administration of LPC (60 μg) had no significant effect (P > 0.05) on the paw withdrawal latency to a 50°C hot plate stimulus.** Data show mean ± SEM for 6 rats/group. Values compared with pre-dose latencies. Click here for file

Additional file 3 **BEL (100 μg i.pl.) had no effect on the reduction in paw pressure (Randall-Selitto) thresholds evoked by prior intraplantar administration of either capsaicin (1 nmole) or α,βMeATP (1 μmole) in lightly restrained rats.** Data show mean ± SEM for 6 rats/group. *** p < 0.001 vs vehicle. Click here for file

Additional file 4 **Application of acetone to the hind-paws evoked responses (paw withdrawal, flicking, licking) in wild-type *Trpa1*^+/+ ^mice but had little effect in *Trpa1*^-/- ^mice. **Intraplantar injection of 25 mg menthol increased the responses to acetone in wild-type *Trpa1*^+/+ ^but not in *Trpa1*^-/- ^mice. Responses to acetone were measured 15 minutes after menthol administration. Data show mean ± SEM for 6 mice/group. ††† p < 0.001 vs untreated paw *** p < 0.001 vs wild-type mice. Click here for file
