# Supplementary figures and images for: The roles of iPLA2, TRPM8 and TRPA1 in chemically induced cold hypersensitivity
Source: Mol Pain. 2010 Jan 21;6:4. doi: 10.1186/1744-8069-6-4 (PMC2822744; doi:10.1186/1744-8069-6-4)

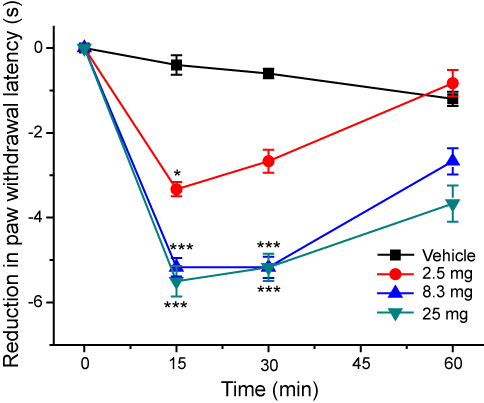

Supplement: Additional file 1 — Intraplantar administration of menthol (2.5-25 mg) induced a time- and dose-dependent reduction in ipsilateral paw withdrawal latency in restrained rats. Data show mean ± SEM for 6 rats/group. * p < 0.05, *** p < 0.001 vs vehicle. [file 1744-8069-6-4-S1.PNG]

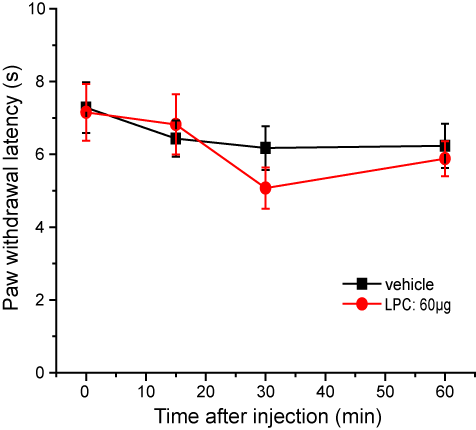

Supplement: Additional file 2 — Intraplantar administration of LPC (60 μg) had no significant effect (P > 0.05) on the paw withdrawal latency to a 50°C hot plate stimulus. Data show mean ± SEM for 6 rats/group. Values compared with pre-dose latencies. [file 1744-8069-6-4-S2.PNG]

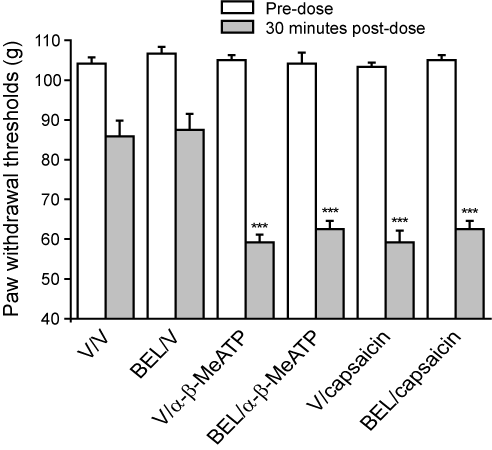

Supplement: Additional file 3 — BEL (100 μg i.pl.) had no effect on the reduction in paw pressure (Randall-Selitto) thresholds evoked by prior intraplantar administration of either capsaicin (1 nmole) or α,βMeATP (1 μmole) in lightly restrained rats. Data show mean ± SEM for 6 rats/group. *** p < 0.001 vs vehicle. [file 1744-8069-6-4-S3.PNG]

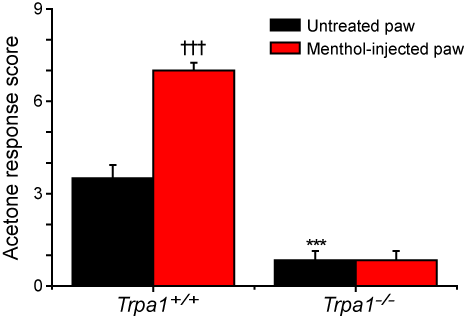

Supplement: Additional file 4 — Application of acetone to the hind-paws evoked responses (paw withdrawal, flicking, licking) in wild-type Trpa1+/+ mice but had little effect in Trpa1-/- mice. Intraplantar injection of 25 mg menthol increased the responses to acetone in wild-type Trpa1+/+ but not in Trpa1-/- mice. Responses to acetone were measured 15 minutes after menthol administration. Data show mean ± SEM for 6 mice/group. ††† p < 0.001 vs untreated paw *** p < 0.001 vs wild-type mice. [file 1744-8069-6-4-S4.PNG]
